# Nanoparticles as an Encouraging Therapeutic Approach to Alzheimer’s Disease

**DOI:** 10.3390/ijms26167725

**Published:** 2025-08-10

**Authors:** Joanna Koga-Batko, Katarzyna Antosz-Popiołek, Hanna Nowakowska, Marta Błażejewska, Eunika Milena Kowalik, Jan Aleksander Beszłej, Jerzy Leszek

**Affiliations:** 1 Faculty of Medicine, Wroclaw Medical University, Ludwika Pasteura 1, 50-367 Wrocław, Poland; antosz0715@gmail.com (K.A.-P.); ht.nowakowska@gmail.com (H.N.); marta.blazejewska@student.umw.edu.pl (M.B.); eunika.m.kowalik@gmail.com (E.M.K.); 2 Clinic of Psychiatry, Department of Psychiatry, Wroclaw Medical University, Ludwika Pasteura 10, 50-367 Wrocław, Poland; jan.beszlej@umw.edu.pl

**Keywords:** Alzheimer’s disease, dementia, nanoparticles, blood–brain barrier, nanocarriers

## Abstract

Alzheimer’s disease (AD) is an irreversible neurodegenerative disease of the central nervous system, responsible for 60–80% of dementia. Its pathogenesis is mainly based on the accumulation of beta-amyloid and tau proteins. Current pharmacological treatment includes acetylcholinesterase inhibitors, NMDA receptor antagonists, and monoclonal antibodies. However, their effect is limited by the blood–brain barrier (BBB). A new and promising way for different drugs to cross the BBB is the use of nanoparticles such as liposomes, micelles, solid lipid nanocarriers, polymeric nanoparticles, dendrimers, nanoemulsions, and inorganic nanoparticles as their carriers. Additionally, some nanoparticles present anti-inflammatory or neuroprotective effects. Some of them can also be used to treat cerebral amyloid angiopathy (CAA) by aiming at amyloid deposits in brain arterioles. All the properties of nanoparticles listed and discussed in the article allow us to hope that there will be more effective treatment in the future, which is extremely important as the number of patients with AD is still growing.

## 1. Introduction

Alzheimer’s disease (AD) is an irreversible, age-related, slowly progressive, and devastating neurodegenerative disease. AD is the main cause of dementia and, at present, millions of people worldwide are affected by this neuropathology. As human life expectancy rises and the prevalence of AD increases with age, the number of people living with AD is growing which is becoming a significant public health challenge [1,2,3,4,5].

The current treatment for AD is the use of cholinesterase inhibitors and N-methyl-D-aspartate receptor antagonists. Unfortunately, this is only a symptomatic solution that does not stop or delay the progression of the disease. The first disease-modifying therapies for AD are two anti-amyloid monoclonal antibodies—aducanumab and donanemab [1,6].

The challenge is to deliver the medicine directly to affected brain regions. Difficulty, inter alia, lies in crossing the blood–brain barrier (BBB). The BBB is built with endothelial cells, pericytes, astrocytes, and the basement membrane. Its function is to protect the central nervous system and regulate brain homeostasis. The problem of drug delivery through the BBB can be solved by nanotechnology—the use of nanoparticles (NPs) as smart drug distribution systems [3,7,8].

This paper explores the classification and characteristics of NPs. In addition, we will discuss the potential use of NPs in the treatment of AD.

## 2. Alzheimer’s Disease—Basic Facts

Alzheimer’s Disease (AD) is a neurodegenerative disease of the central nervous system and the major cause of dementia worldwide—it is responsible for 60–80% of its cases. As the population of people aged 65 and above is growing, it is estimated that the number of patients with AD will also increase [9]. 

The pathogenesis of AD is based primarily on the accumulation of beta-amyloid (Aβ) into plaques that form outside the neurons and the formation of tau tangles inside the neurons. Both those processes are mainly responsible for interference with transmission between the synapses and blockage of the transportation of nutrients and molecules that nourish neurons. The presence of those toxic proteins also leads to the activation of microglia, which are immune system cells present in the brain, and pro-inflammatory cytokines which cause chronic inflammation. In the course of AD, accelerated atrophy of the brain volume is also observed [9,10]. An important part of the AD pathogenesis is also the degeneration of the cholinergic neurons in the nucleus basalis of Meynert [10]. Current studies have also found that an important part in AD’s pathogenesis is played by vascular pathology and age-related changes in the blood vessels, including brain–blood barrier (BBB) dysfunction, hypoperfusion, and hypoxia [11,12]. Early AD can also be impacted by mitochondrial malfunction, which results in higher ROS production, increased oxidative stress, and lower energy production [13,14].

The main signs of AD are memory loss disrupting daily life, difficulty in completing tasks, planning or solving problems, confusion with time and place, appearance of new problems with speaking, writing, and understanding visual images. Patients with AD also experience withdrawal from social activities, work, and their mood, and their personality or behavior may change; they become confused, anxious, suspicious or depressed, which makes it difficult for them to be independent in their daily activities. The disease might also affect other systems and lead to troubles in walking, swallowing or eating. AD progresses in time from preclinical AD, mild cognitive impairment due to AD, to Alzheimer’s dementia [9,14].

The risk factors of AD are, among others, age. The prevalence of AD increases with age; it affects 5.0% of people aged 65 to 74, 13.2% of people aged 75 to 84, and 33.4% of people aged 85 and older. Other risk factors for AD include: genetics—APOE-e4, Down syndrome, genetic mutations of the APP gene, genes for presenilin-1 and presenilin-2, and a first-degree relative with AD. Modifiable risk factors such as obesity, high cholesterol levels, smoking, hypertension, low physical activity, cerebral hypoperfusion, cerebrovascular injury, stroke, severe head trauma or traumatic brain injury, low cognitive reserve, and diet also contribute to the development of AD. However, there are also some factors that decrease the possibility of AD. These include the following: formal education and remaining socially and mentally active throughout life, a heart-healthy diet (Mediterranean, Dietary Approaches to Stop Hypertension—DASH, or Mediterranean–DASH Intervention for Neurodegenerative Delay—MIND), and physical activity [9,14,15].

The diagnostic process of AD can be based on ICD-11 criteria, which include meeting all the diagnostic requirements for dementia, the presumption of AD based on clinical assessment, standardized neuropsychological and cognitive testing, neuroimaging, genetic and medical tests, family history, gradual onset with progressing memory, word-finding difficulties, mild functional impairment [16]. The diagnosis can also be made based on the DSM-5 criteria. Those include meeting the requirements of at least mild neurocognitive disorder, insidious onset, gradual progression of at least one cognitive disorder. Probable AD is diagnosed when there is evidence of a causative AD genetic mutation or all of the following are present: evidence of a decline in memory and learning and at least one other cognitive domain, gradual decline in cognition, and no evidence of mixed etiology [17]. An indispensable part of the diagnostic process is the exclusion of other causes of dementia such as vitamin B12 deficiency, hypothyroidism, HIV, or depression [15]. Currently, the use of additional tests, such as TK, MRI, FDG amyloid PET, CSF examination, is also becoming standard. TK and MRI show hippocampal and cortical atrophy in temporal and parietal regions. The changes observed in CSF include low Aβ, [14,15,18].

Currently used treatment strategies of AD include both pharmacologic and nonpharmacologic management. Nonpharmacologic interventions and behavioral strategies are the first line option, and they are psychoeducation of both the patient and the caregiver. When it comes to pharmacologic options, the most commonly used are acetylcholinesterase inhibitors: donepezil, rivastigmine, and galantamine, which improve cognition, NMDA receptor antagonist—memantine, used in moderate and severe forms of AD. There are also more modern therapies targeting Aβ, and they involve monoclonal antibodies, such as aducanumab and donanemab [2,10,15]. Many scientists are working on other possible targets and molecules that could be treatment options for AD, as it is a significant problem worldwide. Our paper concentrates on the possible use of nanoparticles in the course of treatment of AD.

## 3. Nanoparticles

### 3.1. The Basics of NPs and Nanotechnology

As this paper aims to highlight the possibilities of using NPs in the therapy of AD, it seems crucial to portray the basics of nanotechnology and the NP-transmission system.

Nanotechnology has been developing since the 1990s, and over the decades, and especially in the last few years, it has evolved strongly. Thanks to new technologies and optimized delivery systems, more and more ideas and patents have been introduced into clinical trials and have been approved by the Food and Drug Administration (FDA) or European Medicines Agency (EMA) [19].

Nanotechnology, from its definition, is focused on the matter in the 1 to 100 nm dimension range. This technology provides exceptional benefits that cannot be obtained using materials and systems on a larger scale. The ultra-tiny sizes go hand in hand with an enormous surface area and high reactivity. Since the interaction conditions and laws that apply to such small particles are very different from those in natural, bulk dimensions, the physical and biomolecular characteristics of NPs are also significantly different [20,21]. These diverse properties can sometimes be the answer to the problems and difficulties faced by standard drugs, as it is possible to modify the most elementary characteristics, such as solubility, diffusivity, blood circulation half-life, drug release characteristics, and immunogenicity [20]. Additionally, dedicated NPs can act biocompatible with natural systems, which are also built on a nanoscale. They can create effective drug delivery systems with special targets, like tumors or other tissues characteristically changed by the disease [21,22].

Moreover, in recent years, the true peak of nanotechnology potential has been demonstrated by the massive, worldwide use of lipid nanoparticle mRNA vaccine against COVID-19. Such a large pool of data and vast research brilliantly underscores the enormous potential of these particles, which can potentially transfer anything attached to them to their target location [23].

### 3.2. The Classification of NPs

This paragraph is focused on the general properties and features of diverse NPs. Specific examples of potential applications and systems already in use are described later in the work, in the section devoted to the neuroprotective and anti-inflammatory effects of NPs.

**Figure 1:**
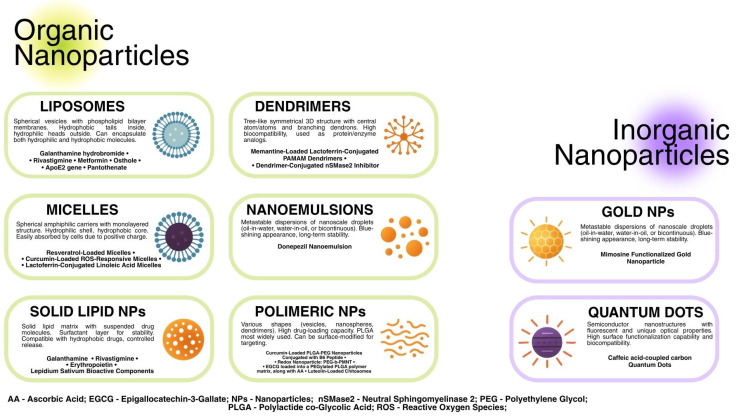
Classification of Nanoparticles in Alzheimer’s Disease treatment.

There are many different ways of categorizing NPs. Variables such as shape, size, and chemical characteristics can be taken into consideration. The main division is based on two categories, which include organic NPs (liposomes, micelles, polymeric, etc.) and inorganic (gold, silica, iron oxide, etc.) [22] [Figure 1].

#### 3.2.1. Liposomes

Starting with the most typical and well-known organic NPs, liposomes are featured as supramolecular aggregates with exceptional amphiphilic characteristics. Although they are polar lipids, they are dispersed in an aqueous solution. Liposomes are built from phospholipid bilayer membranes that form into spherical vesicles. The hydrophobic tails are packed inside the vesicle wall, as the hydrophilic heads are located on the outside, in contact with the surroundings, and the very center of the structure, in contact with the interior space. The described special inner space and lipid walls enable the encapsulation of both hydrophilic and hydrophobic molecules [1,24,25,26,27].

It is also worth highlighting that liposomes are relatively easy to obtain and offer the possibility of a high degree of customization of properties (such as size, composition, or degree of lamellarity) [24,27]. Their applications are invariably wide. They can constitute a delivery system for a huge range of molecules requiring special transport conditions, or those that cannot, by themselves, overcome the barriers that an amphiphilic molecule can overcome. Liposomes are used as components of medicinal preparations, vaccines, or particles enabling imaging studies [24,26,28].

#### 3.2.2. Micelles

Moving further on the topic of organic structures, micelles are worth saying a few words about. Micelles are also characteristic of spherical amphiphilic drug carriers in the nanoscale of the 5–50 nm range. The difference is in the monolayered structure and position of the heads and tails of phospholipids, as the shell is hydrophilic and the core is hydrophobic. It turns out that despite the slightly negative charge of the cell membrane, positively charged NPs such as micelles can be easily absorbed by cells [1,29]. The penetration speed of such particles is even higher than that of charge-neutral particles. Micelles seem to be more attractive to the epithelium, which absorbs them more readily and favors the transport of the molecules contained in them [30,31,32].

#### 3.2.3. Solid Lipid NPs

Another lipid-based structure may be larger than the particles already discussed. Solid-state nano lipids, also known as Solid Lipid NPs (SLNs), are spherical systems estimated to be 50 to 1000 nm in diameter. SLNs consist of a solid lipid matrix with suspended molecules, especially drugs. Moreover, the layer of surfactant is necessary to stabilize the structure in an aqueous solution. SLNs can be applied in very different ways, such as oral and parenteral, transdermal, intranasal, ocular, or directly to the respiratory system. SLNs feature high safety, bioavailability, and overall therapeutic effects [33]. The solid-state systems are perfectly compatible with hydrophobic drugs, and furthermore, they provide a controlled release of these drugs. To increase drug permeation, some specially dedicated upgrades are being created, for example, certain lipids and surfactants may present features of P-glycoprotein inhibitors, or the surface of SLNs can be coated by particular polymers, such as chitosan [33,34,35]. From recent studies, it turns out that thanks to these properties and all the ways of maneuvering them, SLNs appear to provide an ideal way of transporting drugs across the BBB to the specific target sites in the brain [1,36].

#### 3.2.4. Polymeric NPs

Polymeric NPs (PNPs) are another group to discuss. However, they are also partly described in the above groups, as polymeric structures can take on different shapes, such as polymeric vessels, nanospheres, dendrimers, polymeric micelles, and polymeric hydrogels. Their size within the size ranges from 1 to 1000 nm, and the possibility of being covered with dedicated coats, allowing for targeted transport, makes them already widely used drug carriers. In addition to the basic advantages of their nano size, these particles are also characterized by high drug-loading capacity. Additionally, it is worth mentioning the building blocks of PNPs, which can be natural or synthetic biomaterials, including chitosan, albumin, gelatin, collagen, polylactic acid, polylactide co-glycolic acid (PLGA), polylactic acid (PLA), polyethylene glycol (PEG), and polycaprolactone (PCL). From those described, PLGA is the most widely investigated one with a great set of features. The low toxicity, biocompatibility with various cells, and convenient properties of sustained-release explain its long list of applications already created, also in AD [37,38,39].

#### 3.2.5. Dendrimers

The Greek word “dendron” means tree, meros, or branch, and it is a great analogy for dendrimers, which comes from there [40]. Dendrimers are built in a quite elegant, symmetrical way. The center of the structure is an atom or atoms around which the branches spread. Branches, also known as dendrons, create a specialized, well-defined 3D spherical structure. They act with high compatibility on the biological system response for their wide range of applications including medical ones, where they are used as analogs for proteins, enzymes, or viruses to create a desired response of the system [40,41].

#### 3.2.6. Nanoemulsions

Nanoemulsions (NEs) are the last of the organic NPs discussed. NEs are metastable dispersions of nanoscale droplets of one fluid within another fluid. There are three main types of emulsions: oil-in-water, water-in-oil, and bicontinuous NEs, in which oil and water microdomains are dispersed throughout the system. Referring to their physicochemical features, NEs can be placed between microemulsions and traditional ones. These ultra-tiny emulsions of droplet size are in the range of 20–500 nm and are characterized by their peculiar blue-shining appearance. Furthermore, their nano-size prevents creaming, which means efficient long-term stability. Therefore, NEs are used in many fields, e.g., cosmetics, food products, pharmaceuticals, and the petroleum industry. A potential downside to NEs could be the high energy input to prepare them through the process of high-pressure homogenization [42,43].

#### 3.2.7. Inorganic NPs

Last, but not least, the inorganic NPs should be mentioned. The most broadly used inorganic NPs include pure metals, such as Gold and Silver, metal and metalloid oxides (e.g., SiO_2_, γ-Fe_2_O_3_/Fe_3_O_4_), calcium phosphates, or semiconductor materials (Quantum dots). These have already been successfully used, among others, in the cosmetics industry or as a tool in bioimaging for disease diagnosis (Silver NPs (AgNPs), iron oxide NPs (IONPs), and titanium dioxide NPs (TiO_2_NPs)) [C].

Recently, the issue of an additional possibility in the form of nanocarriers for reaching the CNS has been raised. Inorganic NPs, such as gold NPs (AuNPs) and silica NPs (SiO_2_ NPs), seem to be a great alternative to organic ones, as their level of functionality is far more flexible and versatile. This group is very broad, but the focus is on its common features [44,45,46]. Specific examples of inorganic NPS will be presented in paragraph 6. As a typical example of this group, AuNP will be briefly discussed. AuNP is extensively studied, as it acts with a high level of biocompatibility, is easy to synthesize or bioconjugate, and indicates great physical properties such as thermostability. It is commonly used, e.g., in chemotherapy. As described later, quantum dots (QDs), the semiconductor NPs, have fluorescent and unique optical properties that can be useful in the field. However, these particles have significant toxic properties, and this applies not only to AuNP or QDs but to the entire inorganic group in general. The cytotoxicity is strongly connected with the shape, size, concentration, redox activity, chemical stability, surface coating, and others. There are reports that these particles cause insomnia, vertigo, hypokalemia, lymphopenia, or neurodegenerative diseases. However, the mechanisms responsible for their cytotoxicity remain questionable [47,48].

### 3.3. Importance and Possibilities in AD Treatment

The challenges of treating AD often relate to estimating the ideal dose needed, limiting side effects, or ensuring that a given substance reaches the desired site of action in the highest local concentration possible. Since the center of the pathogenesis of AD takes place in the nervous system surrounded or rather protected by a specially selected barrier (BBB), appropriate systems delivering substances are desirable in improving therapy. Nanotechnology may be the answer to the challenges presented, as it enables the manipulation of complex biosystems with greater selectivity and timing than conventional drugs. This direction seems to be mostly required in terms of BBB and penetration through it. NPs, as discussed in this paper, seem to be, both in theory and in the research, an idea worth considering for the challenges presented [7,49].

## 4. Nanotechnology-Based Drug Delivery Systems Through BBB in AD

BBB is a homeostatic defense mechanism that protects the brain against pathogens and neurotoxins circulating in the bloodstream. It also provides ionic homeostasis, brain nutrition, and regulates the level of neurotransmitters. However, it is not completely impermeable as it allows the passage of desired molecules into the brain parenchyma. Due to its existence intracerebral administration of drugs is limited [50,51,52].

The integrity of BBB is provided by the cells that are its components. Those are: endothelial cells, pericytes, and astrocytes [52].

Endothelial cells in cerebral capillaries are characterized by the expression of tight junctions that prevent unregulated passage of molecules that are water-soluble between the endothelial cells and separate their luminal portion from the basalis region. What sets them apart from other vascular endothelia is also the lack of pinocytic activity, fenestrations, the expression of active transport mechanisms and the presence of drug-metabolizing enzymes. There is a free diffusion of oxygen, carbon dioxide, and small lipophilic molecules through the endothelium [52].

Pericytes are connected to endothelial cells via N-cadherin and connexins. Those connections allow the exchange of ions, metabolites, second messengers, and ribonucleic acids. Pericytes are also responsible for microvascular stability, they also help with the removal of toxic metabolites and play a vital role in cerebral autoregulation [52,53].

Astrocytes are responsible for various tasks in the CNS, including maintaining the ionic hemostasis, pH regulation, compartmentalization of neural parenchyma and the mediation of signals. They therefore play a vital role in maintaining the barrier function of the endothelial cells of the brain [52,54].

The transport across the BBB occurs through various forms of transport including passive transcellular diffusion (lipid-soluble small molecules), paracellular diffusion (water-soluble small molecules), active efflux (via ATP-binding cassette proteins), carrier-mediated transport (e.g., amino acids, carbohydrates, fatty acids, nucleotides, amines, vitamins), receptor-mediated transport (e.g., regulatory, proteins, hormones, growth factors, neuroactive peptides), and absorptive-mediated transport [52,55]. Although it is possible for drugs to enter the brain via the mentioned pathways, there are still several conditions that the drug has to fulfill to be able to penetrate the BBB. Those include molecular weight lower than 400Da, spherical shape, physiological pH value, physiological pH value or lipophilicity. Thus, most of the drugs cannot cross the BBB efficiently, which impacts their bioavailability and challenges scientists when it comes to effective treatment strategies of many diseases, including AD [56,57]. This is why there are several approaches that are trying to bypass this obstacle: local sustained release, which is highly invasive, intranasal administration, and systemic administration with the use of nanoparticle technology [55,56]. Some of the non-invasive approaches are chemical structure transformation, lipidazation, which is the creation of lipophilic analogs using viral vectors or colloidal drug carriers. Invasive methods include intracerebral implants, intrathecal, interstitial or intraventricular delivery. There are also some strategies enabling them to disrupt the BBB: convection-enhanced delivery (CED), osmotic, biochemical, ultrasound-mediated strategies [58]. Our paper focuses on the use of nanoparticles and thus this approach will be further discussed.

Most of the drugs effective in the treatment of AD are characterized by low water solubility and their uptake through BBB is restricted by P-glycoprotein and breast cancer resistant protein (BCRP) [55,57]. A promising strategy to improve their uptake through BBB is using nanotechnology-based drug delivery systems (DDSs). Their size, surface properties and architectures can be modified to increase their effectiveness. They can encapsulate and carry the therapeutic agents through BBB by opening the tight junctions, causing local toxic effects leading to increase in BBB permeability, via endocytosis or transcytosis by interacting with various receptors and transporters on the BBB. They also tend to protect the therapeutic agents and increase their bioavailability. Their indisputable advantages also have low immunogenicity, biocompatibility, long circulation time, enhancing drug absorption, high drug loading, and an ability to target certain molecules or cell types [55,56,59,60].

There are currently various types of nanoparticles (NPs) available: polymeric NPs, lipid-based NPs, inorganic NPs.

Polymeric NPs are characterized by the size of 10–1000 nm, low toxicity and biocompatibility. They consist of a reservoir and matrix system. Their role is to protect the drug from external degradation and control its release. They also transport the encapsulated therapeutic agent through the BBB by passive or active delivery and boost the BBB penetration. All those features result in higher effectiveness of the carried drug [55,56,60,61,62].

Lipid-based NPs include mainly liposomes, nanostructured lipid carriers, and nanoemulsions [63]. They form structures of size varying from 80 nm to 100 μm. They can be loaded with various therapeutic agents as they have an aqueous core that can hold hydrophilic molecules, and a lipid bilayer that can transport particles that are lipophilic. Furthermore, their structure is compatible with the lipid layer of BBB, which makes it easier for them to enter the brain. They are also characterized by good biocompatibility and high safety which makes their use in treating AD highly promising [55,56,61,64].

Inorganic NPs include mainly metal-based NPs. Their size varies from 1 to 200 nm, and it can be modified during the synthesis process. Metals used in metal-based NPs are, for example, gold, silver, silica or titania. They are characterized by an easy-to-control structure, a large surface, magnetism, and optique properties and high chemical and physical stability; however, they can also present long-term immunological reactions, toxicity, and low biocompatibility, which makes them less popular than polymeric or lipid-based NPs [55,56].

To sum up, NPs are a very promising field for scientists to explore when it comes to creating DDSs for medications used in AD. Specific examples of drugs and substances useful in AD that can be transported by NPs will be described in more detail in the following sections of our paper.

## 5. Possible Use of NPs in Vascular Pathology

Nowadays, many researchers draw attention to the connection between AD and vascular pathology.

De la Torre et al. presented the perspective that AD is mainly vascular disorder firstly in 1993, suggesting the crucial role of reduced cerebral blood flow and changes in capillaries in AD pathogenesis [65].

### 5.1. Structural Changes in Vessels in AD

Many studies report changes in vascular basement membrane (VBM), including its thickening and composition changes like shifts in the number of specific proteins such as collagen IV, collagen I, laminin or fibronectin. Kiuchi et al. suggested that some of them, for instance collagen IV and laminin, may induce AB deposition [66]. It may explain the fact that VBM remodeling is reported even before Aβ deposition, which makes it an early marker of AD [67,68].

In their review, Fisher et al. described cerebrovascular pathology common in AD and divided them into two groups: cerebral amyloid angiopathy (CAA) and non-amyloid small vessel disease (NA-SVD). In CAA, Aβ deposits can be found not only in the brain parenchyma, but also in arterioles’ walls, especially in leptomeningeal vessels. It affects about 80% people with AD and only 30–40% elderly without dementia, which emphasizes its correlation [69]. Allen et al. described four different types of AD, depending on Aβ deposition patterns as senile plaques or with vascular domination based on the immunostained sections of 134 AD cases. Although this research showed different pathological presentations, the phenotype and disease manifestation did not vary [70].

In turn, NA-SVD is a term correlated with arteriolosclerosis: collagenous thickening of vessel walls and narrowing of their lumen, especially in small perforating arteries. Changes are described mainly as a consequence of hypertension, although studies also consider the role of a vicious cycle between hypertension and NA-SVD. In this conception, increased cerebrovascular resistance leads to reduced cerebral blood flow, which induces cerebrovascular response and hypertension [71]. Arvanitakis et al., in their cross-sectional study, showed the association between severity of large cerebral vessel (atherosclerosis) or small vessel (arteriolosclerosis) disease and higher AD prevalence [72].

### 5.2. Monoclonal Antibody IgG4.1 Conjugated to Iron Oxide Nanoparticles Monodisperse γ-Fe_2_O_3_ Nanoparticles (MIONs) as Potential Treatment for CAA

Poduslo et al. presented monoclonal antibody IgG4.1, raised against human fibrillar Aβ42 targeting successfully amyloid plaques in AD transgenic mouse brain [73]. IgG4.1 binds specifically to the N-terminal of the Aβ peptide with preference to fibrillar Aβ40 over monomeric Aβ40 peptides. In the study, researchers demonstrated the ability of the IgG4.1-nanoparticles to target CVA deposits in the brain arterioles of transgenic mice brains and detected the presence of these nanoparticles MR-contrast agents using standard MR imaging techniques. Although these results are restricted only to brain regions with no BBB, CAA mainly refers to leptomeningeal fenestrated vessels that enable particles to reach pathological deposits. Targeted nanoparticles give future opportunities for both diagnosis and treatment of CAA. It is worth mentioning that CAA impedes the cerebral penetration of other therapeutic agents so its reduction would expand AD treatment possibilities.

### 5.3. Common Risk Factors for AD and Cardiovascular Disorders

The following argument for the possible vascular pathogenesis of AD, as widely discussed in many studies, is common risk factors of cardiovascular disease and AD. Association between type-2 diabetes and dementia, including AD, is well-established, although the causal nature of this state is not obvious [74,75]. Relation with other risk factors is uncertain and seems to be more complicated. Some evidence shows that high body weight increases the risk of AD [76,77]. On the other hand, Tolppanen et al. presented that obesity in mid-life is associated with an increased risk of AD, whereas high body mass index in the elderly seems to have protective effect [78]. Interestingly, underweight in middle age is reported to be correlated with a higher risk of dementia and AD as well [76]. Evidence that diet, smoking, hypercholesterolemia, hypertriglyceridemia or the statins use may impact AD progression tend to be contradictory and, despite the great amount of studies related to those topics, no correlation seems to be confirmed so far [79,80,81].

### 5.4. Role of Endothelin-1 and Angiotensin System as the Potential Therapeutic Targets

Pathology widely described in AD is cerebral hypoperfusion. It is not only observed in the brain affected by AD but even precedes dementia onset and evidence shows that rather exacerbates brain atrophy than is a consequence of reduced metabolism connected to neuronal loss [82,83,84,85]. These changes may be detected in imaging studies and by measuring hypoperfusion markers, such as the ratio of myelin-associated glycoprotein (MAG) to proteolipid protein-1 (PLP1) [85,86]. Increasing evidence suggests that changes in cerebral blood flow, especially in the early stage of AD, are rather non-structural and depend on dysregulation in vasoactive mediators’ production [82,85]. It includes an increased amount of endothelin-1 (ET1), a strong vasoconstrictor. Its production is catalyzed by endothelin-converting enzymes (ECEs) proved to be upregulated by Aβ (Palmer). Endothelin1-receptors antagonists, like bosentan, which are successfully used in pulmonary hypertensive therapy, are nowadays tested as the treatment opportunity in AD [87,88]. However, bosentan presents low bioavailability due to the low water solubility which may be potentially solved by use of nanoparticles. Production of bosentan nanoparticles and nanosuspension that aims to utilize as pulmonary hypertension treatment has already been reported in studies [89,90,91] but has not been tested in AD so far.

Another treatment option examined intensively is connected with the renin–angiotensin system (RAS). In their review, Kehoe et al. have summarized the effects of angiotensin, angiotensin-converting enzyme (ACE), and the role of its pharmacological suppression in AD, as observed in vitro and in vivo. Researchers draw attention to unsolved doubts about the RAS mechanism of action in AD and problems connected with trials on patients, such as group selection, comorbidity, and drug penetration to the brain. Nevertheless, inhibiting RAS by widely used antihypertensive treatments like ACE-inhibitors (ACEI) or angiotensin-receptor blocker (ARB) seem to offer many benefits for AD therapy [92]. The following trials suggest ARB to be dominant over ACEI in preventing dementia [93,94]. Some of these drugs penetrate through BBB but the use of NPs combined with them may prevent their systemic action. RAS-related nanoparticles have already been produced for different treatment purposes but so far none have been studied in AD [95].

### 5.5. Natural Substances Use in AD Therapy

It is worth mentioning that AD treatment options include natural compounds, such as berberine or quercetin, substances which are widely used in traditional Chinese medicine [96,97]. Nowadays, they are studied, specifically in that region, and much evidence reports their beneficial action in many clinical problems, for instance atherosclerosis, cardiovascular diseases, and metabolic disorders [96]. In their meta-analysis, Dan et al. summarized studies about the use of berberine in AD. According to them, berberine may be a potential treatment for dementia; however, the results are limited due to the quality of available data and more rigorous research is necessary to confirm this [98]. The direct mechanism of berberine action is unclear but suggested methods include anti-inflammatory and anti-oxidative activity as well as reducing potential additional triggers like atherosclerosis or metabolic pathologies [96,98,99]. To improve drug penetration to the nervous system, Singh et al. synthesized lipid-coated mesoporous silica nanoparticles (MSNs) containing berberine, which presented better activity compared to a pure substance [100]. Abo El-Enin et al. produced berberine-laden nanostructured lipid carriers overlaid with chitosan, which may be administered intranasally with good pharmacokinetics and brain uptake [101]. However, the utilization of these substances is limited due to a lack of sufficient data from clinical trials.

## 6. Neuroprotective and Anti-Inflammatory Effects of Nanoparticles

The etiopathogenesis of Alzheimer’s disease (AD) presents a significant epidemiological burden, with current therapeutic interventions limited to symptomatic management rather than disease modification. The neuropathological hallmarks of AD are characterized by two primary proteinopathies: extracellular beta-amyloid (Aβ) peptide aggregation forming senile plaques, and intraneuronal accumulation of hyperphosphorylated tau protein manifesting as neurofibrillary tangles (NFTs). This neurodegenerative disorder exhibits progressive deterioration through complex cascades of molecular and cellular dysfunction, leading to significant alterations in neuronal architecture and synaptic plasticity throughout the central nervous system (CNS) [1,3,102,103,104].

Contemporary therapeutic research strategies for AD prioritize multi-target approaches that address interconnected pathophysiological pathways implicated in its etiology. A fundamental challenge in neurodegenerative disorder therapeutics lies in developing targeted drug delivery systems capable of crossing the BBB and achieving optimal CNS biodistribution. Nanotechnological approaches present a promising solution to these constraints. NPs—specifically engineered biocompatible materials loaded with therapeutic compounds, facilitate enhanced BBB penetration, and targeted neuronal delivery, potentially mitigating neurodegeneration while demonstrating superior safety profiles compared to conventional therapeutic methods [4,105,106].

To provide a structured overview of the diverse therapeutic applications of nanoparticle systems in Alzheimer’s disease treatment, Table 1 presents a comprehensive summary of the various nanocarrier platforms discussed in this review. This table serves as a framework for understanding the detailed discussions that follow.

In the following, we would like to focus on specific examples of research that relates to the NPs forms discussed in paragraph 3, highlighting their neuroprotective and anti-inflammatory properties in the context of AD treatment. The division we have chosen is based on the forms of construction of NPs due to the clarity and possibility of relating paragraphs 3 and 6 to each other.

### 6.1. Liposomes

#### 6.1.1. Currently Approved AD Therapeutics Encapsulated in Liposomes

One of the proposed hypotheses for the etiology of neurodegeneration in Alzheimer’s disease (AD) is the damaging action of free radicals, such as superoxide anion and nitric oxide (NO). Galanthamine hydrobromide (GH) has been shown to protect cholinergic neurotransmission through the inhibition of the enzyme acetylcholinesterase (AChE), enhancing memory and improving cognitive function. Studies have demonstrated that the intranasal (IN) administration of GH-loaded flexible liposomes exhibits superior pharmacokinetic properties—higher Cmax, shorter Tmax, and a significantly enhanced efficiency in inhibiting acetylcholinesterase activity within the rat brain [107].

Studies by Yang et al. demonstrated that cell-penetrating peptide-modified liposomes (CPP-Lp) enhanced transcellular transport across murine brain microvascular endothelial cells in vitro. IN delivery of rivastigmine-loaded liposomes showed superior drug biodistribution in the hippocampus and cortex—key regions in AD pathology—compared to intravenous administration. The formulation exhibited delayed but sustained cholinesterase inhibition, correlating with extended tissue retention. These findings indicate that CPP-modified liposomal rivastigmine enhances brain drug delivery through both blood–brain barrier penetration and direct nose-to-brain transport [108].

#### 6.1.2. Metformin Encapsulated in Phosphatidylserine-Based Liposomes

Evidence suggests that Aβ aggregation in AD triggers microglial activation, leading to neuroinflammatory cascades and oxidative stress-mediated neuronal damage [141]. Phosphatidylserine-functionalized nanoliposomal metformin (MET-PSL) demonstrates potential therapeutic efficacy in mitigating AD-associated cognitive deficits and neuroinflammation. In AD-induced rat models, intraperitoneal administration of MET-PSL demonstrated statistically significant improvements (*p* < 0.05) in cognitive parameters compared to metformin or phosphatidylserine liposomes alone. Furthermore, MET-PSL treatment significantly reduced (*p* < 0.05) hippocampal pro-inflammatory cytokine levels, including interleukin-1β (IL-1β), tumor necrosis factor-α (TNF-α), and transforming growth factor-β (TGF-β). Histopathological analysis revealed a marked reduction in neuroinflammation and neuronal necrosis, concurrent with significantly enhanced (*p* < 0.05) neurogenesis in MET-PSL-treated subjects [109].

#### 6.1.3. Transferrin-Modified Osthole PEGylated Liposomes

Osthole (Ost), a coumarin derivative, demonstrates neuroprotective effects against Aβ oligomer-induced toxicity in hippocampal neurons and neural stem cells, presenting therapeutic potential for AD. Nevertheless, its clinical efficacy is limited by poor aqueous solubility, low bioavailability, and insufficient BBB penetration [142]. To address these limitations, transferrin-modified osthole liposomes (Tf-Ost-Lip) were developed to enhance bioavailability and brain targeting.

In vitro studies demonstrated enhanced cellular uptake of Tf-Ost-Lip in human brain endothelial cells (hCMEC/D3) and amyloid precursor protein-transfected neuroblastoma cells (APP-SH-SY5Y), with improved BBB penetration and cytoprotective effects. Pharmacokinetic evaluation in APP/PS-1 transgenic mice showed prolonged circulation time and enhanced brain accumulation of Tf-Ost-Lip, correlating with the amelioration of AD-associated pathological manifestations compared to unmodified osthole [110].

#### 6.1.4. Surface-Modified Liposomes for ApoE2 Gene Delivery

Arora et al. developed dual-functionalized liposomal systems for the targeted delivery of ApoE2-encoding plasmid DNA (pApoE2) to address AD pathology. The strategy leverages ApoE2’s neuroprotective properties, as this isoform reduces AD risk by 50% and facilitates toxic amyloid-beta clearance while delaying disease onset, in contrast to the pathogenic ApoE4 isoform [111]. The liposomes were surface-modified with mannose (MAN) for glucose transporter-1 (GLUT-1) targeting and either rabies virus glycoprotein (RVG) or penetratin (Pen) as cell-penetrating peptides to enhance brain-targeting, cellular internalization, and blood–brain barrier penetration. These dual-functionalized formulations demonstrated superior transfection efficiency in brain endothelial cells, neurons, and astrocytes compared to mono-functionalized or unmodified liposomes, with approximately two-fold higher protein expression in an in vitro BBB model. In vivo studies in C57BL/6 mice confirmed enhanced brain-specific gene delivery and expression following intravenous administration, without apparent toxicity [112].

#### 6.1.5. Pantothenate-Encapsulated Liposomes

Mendelian randomization analysis revealed pantothenate’s significant prophylactic potential in AD, particularly through its inhibitory effect on Pyruvate Kinase (PKM2) nuclear translocation within microglial cells, which is supported by previous findings of diminished vitamin B5 (pantothenate) levels in inflammatory neurological conditions [114]. This led to the development of transferrin-modified liposomal nanoparticles encapsulating pantothenate (Pan@TRF@Liposome NPs). These nanocarriers demonstrated effective blood–brain barrier penetration and modulation of chromosome region maintenance 1 (CRM1)-mediated PKM2 nuclear translocation in microglia, a critical pathway in AD pathology. The formulation exhibited remarkable biocompatibility and stability, while significantly reducing neuroinflammatory markers (TNF-α, IL-6, IL-1β) and neuronal apoptosis. When combined with exercise, Pan@TRF@Liposome NPs showed synergistic effects in AD animal models, resulting in enhanced cognitive performance and improved neurofunctional outcomes. This dual-modality approach, targeting both metabolic dysfunction and neuroinflammation, presents a promising therapeutic strategy for AD treatment [113].

### 6.2. Micelles

#### 6.2.1. Resveratrol-Loaded Neuronal Mitochondria-Targeted Micelles

Yang et al. developed a novel dual-targeting micellar delivery system (C3 peptide and triphenylphosphonium-modified nanomicelles, CT-NM) for resveratrol—a potent sirtuin 1 (SIRT1) activator with antioxidant, anti-inflammatory, and neuroprotective properties—to address mitochondrial dysfunction in Alzheimer’s disease (AD). Despite resveratrol’s potential to improve mitochondrial function through SIRT1-mediated activation of peroxisome proliferator-activated receptor-gamma coactivator 1α (PGC-1α), its therapeutic efficacy has been limited by poor oral bioavailability and inadequate brain accumulation [143]. The nanocarriers were engineered using poly(ethylene glycol)-b-poly(L-lactide) (PEG-PLA) and functionalized with neural cell adhesion molecule (NCAM) mimetic peptide C3 for neuronal targeting and triphenylphosphonium (TPP) for mitochondrial localization. This targeted approach significantly enhanced resveratrol accumulation in neuronal mitochondria compared to conventional delivery methods or single-modified micelles. The resveratrol-loaded CT-NM demonstrated therapeutic efficacy in APP/PS1 transgenic mice by stabilizing mitochondrial fission-fusion dynamics, upregulating sirtuin 1 expression, reducing amyloid deposition and tau hyperphosphorylation, protecting synapses, and inhibiting microglial proliferation. This strategy overcomes the limitations of traditional antioxidant therapies, such as poor mitochondrial targeting and low brain bioavailability, presenting a promising approach for AD treatment through targeted restoration of neuronal mitochondrial function [115].

#### 6.2.2. Curcumin-Loaded ROS-Responsive Micelles

A novel polymeric micelle drug delivery system (ABPEG-LysB/CUR) was developed to address early-stage AD pathology by targeting microglial abnormality and microenvironmental dysfunction. The system leverages increased receptor expression for advanced glycation end-products (RAGE) on the BBB, neurons, and microglia during neuroinflammation, which mediates plasma Aβ influx into the brain and contributes to neurotoxicity and microglial activation [118]. The system comprises three key components: first, a RAGE-targeting peptide (AB) derived from Aβ protein for enhanced brain accumulation; second, an amphiphilic polymer (poly(ethylene glycol) (PEG)-LysB) designed to respond to and scavenge reactive oxygen species (ROS)—a critical early-stage characteristic of AD that precedes amyloid deposition and contributes to microglial activation; and third, curcumin—a hydrophobic natural compound targets Aβ aggregation and demonstrates neuroprotective properties. Curcumin’s therapeutic potential in AD is attributed to its ability to inhibit Aβ aggregation and modulate the inflammatory microenvironment, though its clinical efficacy has been limited by poor bioavailability [119]. This multifunctional delivery system accumulates in diseased regions through an Aβ transportation-mimicked pathway. The micelles demonstrated synergistic effects in APP/PS1 mouse models through reactive oxygen species scavenging and Aβ inhibition, resulting in a normalized oxidative and inflammatory microenvironment, enhanced neuroprotection, and improved cognitive function. This approach uniquely targets early-stage AD pathology before irreversible neuronal damage occurs, offering advantages over conventional treatments that typically focus on late-stage pathologies [117].

#### 6.2.3. Lactoferrin-Conjugated Linoleic Acid Micelles

A novel self-assembled micellar nanoplatform was developed through the conjugation of lactoferrin (LF) and conjugated linoleic acid (CLA). The system exploits lactoferrin’s ability to cross the BBB through LF receptor-mediated transcytosis, as these receptors are overexpressed on brain endothelial cells [121]. CLA, an omega-6 fatty acid with neuroprotective properties, serves a dual purpose: forming the hydrophobic core of the micelles and acting as an encapsulated therapeutic agent. CLA’s therapeutic potential stems from its ability to inhibit μ-Calpain activation and enhance phospholipase A2 expression, thereby preventing Aβ peptide oligomerization and tau protein phosphorylation [122]. The LF-CLA micelles demonstrated enhanced brain biodistribution and sustained drug release at pH 6.8, with no significant toxicity to liver or kidney function. In an aluminum chloride-induced Alzheimer’s disease animal model, the system showed superior therapeutic efficacy compared to the physical mixture of LF and CLA, evidenced by improved cognitive capabilities, reduced brain oxidative stress, decreased inflammation, and apoptosis, lowered acetylcholinesterase activity, and reduced amyloid β peptide1-42 deposition [120].

### 6.3. Solid Lipid Nanoparticles (SLN)

#### 6.3.1. Currently Approved AD Therapeutics Encapsulated in SLNs

In contrast to Li et al.’s liposomal approach [107], Misra et al. developed SLNs as an alternative nanocarrier system for the delivery of the acetylcholinesterase inhibitor GH, demonstrating another strategy for enhanced brain-targeted drug delivery in AD therapy. The formulation, utilizing glyceryl behenate (Compritol) as the solid lipid carrier, was designed using biocompatible and biodegradable components through a scalable production process. These spherical nanoparticles demonstrated sustained drug release properties, achieving over 90% release over 24 h in vitro. Notably, the SLN formulation exhibited approximately two-fold enhancement in bioavailability compared to the free drug. In vivo studies in cognitive deficit rat models showed significant improvement in memory restoration capabilities compared to the naive drug, suggesting the potential of this delivery system to overcome current therapeutic limitations [36].

Nanostructured lipid carriers (NLCs), representing the second generation of lipid nanocarriers, were developed to overcome the limitations of solid lipid nanoparticles (SLNs). While SLNs contain exclusively solid lipids, NLCs incorporate a blend of solid lipids and liquid oils, creating imperfections in the crystal structure that enhance drug loading capacity and prevent drug expulsion during storage [123]. Chauhan et al. developed rivastigmine-loaded NLCs for transdermal delivery in AD treatment, using glyceryl monostearate as the solid lipid and castor oil as the liquid lipid component. The system was evaluated in Albino Wistar rats, demonstrating superior pharmacokinetic properties compared to conventional formulations, with approximately 1.5-fold higher AUC (865.70 ± 5.88 μg/mL/h) than the marketed Exelon^®^ patch (552.17 ± 3.65 μg/mL/h). The NLC formulation exhibited controlled drug release over 72 h, enhanced bioavailability, and improved skin permeation, attributed to the unique matrix structure of solid and liquid lipids [144].

#### 6.3.2. Erythropoietin-Loaded SLNs

Erythropoietin (EPO), a hematopoietic cytokine, demonstrates significant neuroprotective potential in AD through maintaining neuronal survival, regulating neurogenesis, and activating multiple cellular signal modulations that inhibit apoptotic proteins and block TNFα-induced caspase activation [145]. Dara et al. developed EPO-loaded SLNs using glyceryl monostearate. The neuroprotective effects were evaluated in a beta-amyloid-induced AD rat model using male Wistar rats, with treatments administered every other day for six doses. The EPO-SLN formulation demonstrated superior therapeutic outcomes compared to native EPO, as evidenced by improved spatial memory in Morris water maze tests and reduced oxidative stress markers in the hippocampus. Notably, EPO-SLN (2500 IU/kg) showed comparable efficacy to twice the dose of native EPO (5000 IU/kg), while histological examination revealed a significant reduction in amyloid plaque deposition and enhanced neuronal survival in the hippocampal CA1 region. The system effectively decreased the ADP/ATP ratio, lipid peroxidation, and ROS levels while increasing thiol content, demonstrating EPO’s multifaceted neuroprotective mechanisms through antioxidant and anti-apoptotic pathways [124].

#### 6.3.3. SLNs Loaded with *Lepidium sativum* Seed Bioactive Components

Al-Saran et al. developed *Lepidium sativum* (LS) seed extract-loaded SLNs to enhance bioavailability and neuroprotective efficacy in oxidative stress and amyloid-β-induced neurotoxicity. Lepidium sativum, traditionally used as a medicinal plant, contains bioactive compounds including 3-Isoquinolinamine, triterpene esters, and polyphenols that demonstrate significant neuroprotective properties through antioxidant effects and inhibition of β-amyloid-induced apoptosis. The system utilized glyceryl monostearate and hyaluronic acid with chia seed fatty acids. The neuroprotective effects were evaluated using human neuroblastoma SH-SY5Y cells and human mesenchymal stem cells (hMSCs). The LS-SLNp formulation demonstrated superior therapeutic outcomes compared to native LS extract, evidenced by enhanced cell viability and reduced oxidative stress markers. Pre-treatment with LS-SLNp (4 μg/100 mL) effectively protected cells against H_2_O_2_ and Aβ1-42-induced toxicity by increasing mitochondrial membrane potential and reducing apoptotic markers. Moreover, the system significantly upregulated Wnt/β-catenin/Camk-II signaling pathway genes (Wnt3a, Wnt5a, Wnt7a) and decreased neuroinflammatory markers. The enhanced neuroprotective effects were attributed to improved bioavailability and targeted delivery through the dual-functionalized nanocarrier system, suggesting potential therapeutic applications in neurodegenerative disorders [125].

### 6.4. Polymeric NPs (PNPs)

#### 6.4.1. Curcumin-Loaded PLGA-PEG Nanoparticles Conjugated with B6 Peptide

In another study exploring the potential therapeutic effects of curcumin in AD, researchers synthesized poly(lactide-co-glycolide)-block-poly(ethylene glycol) (PLGA-PEG) conjugated with B6 peptide and loaded it with curcumin (PLGA-PEG-B6/Cur) to improve brain bioavailability and targeting. They evaluated the effects of PLGA-PEG-B6/Cur nanoparticles in APP/PS1 transgenic mice. Histological analysis revealed that these nanoparticles significantly reduced Aβ accumulation and tau hyperphosphorylation in the hippocampus. Notably, a three-month treatment with PLGA-PEG-B6/Cur nanoparticles markedly improved spatial learning and memory in APP/PS1 mice compared to treatment with native curcumin. These findings together indicate that PLGA-PEG-B6 can profoundly improve the delivery efficiency of nanoparticles to the brain and that PLGA-PEG-B6/Cur nanoparticles may serve as a promising therapeutic strategy for the treatment of AD in the future [126].

#### 6.4.2. Redox Nanoparticles

Recent investigations have demonstrated that redox nanoparticles exhibit considerable therapeutic potential in AD, particularly due to their capacity to scavenge ROS and mitigate free radical-induced cellular damage. These nanostructures demonstrate significant biocompatibility, extended circulation times, and minimal cytotoxicity, while exhibiting both anti-inflammatory and potential anti-aging properties [128].

Boonruamkaew et al. developed a novel redox nanoparticle (RNPN) composed of an amphiphilic block copolymer, poly(ethylene glycol)-b-poly[4-(2,2,6,6-tetramethylpiperidine-1-oxyl)aminomethylstyrene] (PEG-b-PMNT), which exhibits self-assembly characteristics to form antioxidant polymeric structures. These nanoparticles offer significant advantages over conventional low-molecular-weight (LMW) antioxidants because their size prevents internalization into healthy cells, thereby avoiding disruption of normal redox reactions and electron transport chains. The incorporation of TEMPO (2,2,6,6-tetramethylpiperidine-1-oxyl) moieties in the PMNT segment act as stable radicals that catalytically react with reactive oxygen species (ROS), making them potent antioxidants. In AD investigations, RNPN administration via oral delivery to Tg2576 transgenic mice over six months demonstrated significant neuroprotective efficacy. The study revealed a marked reduction in oxidative stress biomarkers, a substantial decrease in Aβ concentrations (both Aβ1-40 and Aβ1-42 isoforms), and enhanced cognitive performance in spatial and non-spatial memory assessments compared to control subjects. The pH-sensitive nature of RNPNs allows them to disintegrate in the acidic environment of the stomach, enabling absorption through the small intestine and subsequent circulation in the bloodstream, where they can effectively cross the BBB to deliver their therapeutic effects [127].

#### 6.4.3. Epigallocatechin-3-Gallate (EGCG) Loaded into a PEGylated PLGA Polymer Matrix, Along with Ascorbic Acid (AA)

Cano et al. investigated novel nanotechnology-based strategies to enhance the therapeutic potential of epigallocatechin-3-gallate (EGCG), a potent polyphenol from green tea, for AD. Despite EGCG’s multifaceted neuroprotective properties, including inhibiting tau aggregation, reducing Aβ accumulation, and suppressing neuroinflammation, its clinical translation is limited by poor bioavailability and instability [130]. To overcome these challenges, the researchers developed nanoparticles composed of EGCG loaded into a PEGylated PLGA polymer matrix, along with ascorbic acid (AA) to prevent EGCG degradation via its antioxidant activity [131]. When administered orally to APP/PS1 transgenic mice, the EGCG/AA nanoparticles exhibited remarkable therapeutic efficacy. Treatment led to reduced Aβ plaque burden, decreased soluble, and insoluble Aβ (1-42) peptides in the cortex, and diminished neuroinflammation compared to untreated transgenic mice. Notably, the nanoparticles enhanced synaptogenesis and improved spatial learning and memory performance [129].

#### 6.4.4. Luteolin-Loaded Chitosomes

Luteolin, a naturally occurring flavonoid found in various vegetables and fruits, was selected due to its promising therapeutic potential in reducing AD symptoms through its antioxidant and anti-inflammatory properties, as well as its ability to modulate transcription factors and inhibit various protein kinases [133]. However, luteolin’s poor solubility, extensive first-pass metabolism, and low blood–brain barrier permeability necessitated an improved delivery system. Chitosan, a natural cationic polysaccharide derived from chitin deacetylation, was selected as a carrier material due to its unique properties including biocompatibility, biodegradability, and ability to enhance drug permeation across the blood–brain barrier [134]. Using adult Swiss Albino male mice, the researchers demonstrated that intranasal administration of luteolin-loaded chitosan-decorated nanoparticles (chitosomes) significantly improved cognitive function, spatial memory, and learning capabilities compared to free luteolin suspension. The neuroprotective effects were evidenced by increased neuronal survival rate, reduced Aβ plaque formation, and improved antioxidant status through enhanced GSH levels and reduced MDA. The chitosan-based delivery system achieved these effects at lower doses due to its mucoadhesive properties and ability to form electrostatic interactions with negatively charged endothelial surfaces, leading to prolonged retention in the nasal cavity. Additionally, histological evaluation revealed that chitosomes provided superior protection against hippocampal damage and significantly reduced neuroinflammatory markers compared to free luteolin, demonstrating the potential of this delivery system for enhanced brain targeting in Alzheimer’s disease therapy [132].

### 6.5. Dendrimers

#### 6.5.1. Memantine-Loaded Lactoferrin-Conjugated PAMAM Dendrimers

Gothwal et al. investigated the neuroprotective effects of memantine-loaded lactoferrin-conjugated PAMAM dendrimers in an aluminum chloride-induced Alzheimer’s disease mouse model [135]. Memantine (MEM), an NMDA receptor antagonist approved for moderate to severe AD, works by blocking excessive NMDA receptor activation, which leads to excitotoxicity and neuronal death in Alzheimer’s disease [136]. However, its daily administration requirements and poor drug compliance necessitated an improved delivery system. Using Swiss albino mice and Sprague Dawley rats, the research demonstrated that lactoferrin-conjugated PAMAM dendrimers significantly improved MEM’s brain bioavailability and therapeutic efficacy. The nanoformulation achieved sustained drug release over 48 h and showed significantly higher brain uptake compared to free MEM. The neuroprotective effects were evidenced by improved cognitive function and object recognition memory in AD-induced mice, with the discrimination index significantly higher in MEM-PAMAM-Lf treated groups compared to aluminum chloride controls. Importantly, while the formulation did not affect acetylcholinesterase activity or dopamine levels, it demonstrated its therapeutic effect through NMDA receptor-mediated pathways rather than cholinergic mechanisms. This targeted delivery system achieved these effects at lower doses due to lactoferrin’s ability to facilitate drug transport across the blood–brain barrier through receptor-mediated transcytosis [135].

#### 6.5.2. Dendrimer-Conjugated nSMase2 Inhibitor

Tallon et al. explored a novel therapeutic approach for Alzheimer’s disease by investigating the dendrimer-based delivery of 2,6-Dimethoxy-4-(5-Phenyl-4-Thiophen-2-yl-1H-Imidazol-2-yl)-Phenol (DPTIP), a potent inhibitor of neutral sphingomyelinase 2 (nSMase2). This enzyme was targeted due to its critical role in regulating extracellular vesicle release, which facilitates the spread of pathological tau between neurons—a key mechanism in AD progression. Using male transgenic mice, the research demonstrated that dendrimer conjugation significantly improved DPTIP’s pharmacokinetic properties, achieving sustained drug release over 48 h and enhanced brain targeting compared to free DPTIP. The dendrimer-DPTIP conjugate (D-DPTIP) selectively accumulated in areas of neuroinflammation and effectively reduced tau propagation in the brain, showing a reduction in tau spread. This targeted delivery system was particularly effective because hydroxyl-PAMAM dendrimers can selectively localize in activated microglia in diseased brain regions while showing minimal uptake in healthy tissue. Additionally, the dendrimer conjugation enhanced DPTIP’s poor oral bioavailability and limited brain penetration, allowing for more effective treatment at lower doses. The study demonstrated that this approach not only improved drug delivery but also resulted in significant inhibition of nSMase2 activity and reduction in pathological tau spread [137].

### 6.6. Nanoemulsions

#### Donepezil Nanoemulsion

Research conducted by Kaur et al. explored a novel therapeutic approach for treating Alzheimer’s disease through IN delivery of donepezil using nanoemulsion technology. The study was driven by the limitations of conventional oral donepezil administration, which result in poor brain penetration and significant gastrointestinal side effects, despite the drug’s proven ability to enhance brain acetylcholine levels and reduce neuroinflammation [138]. The researchers developed an oil-in-water nanoemulsion composed of labrasol, cetyl pyridinium chloride, and glycerol, achieving a particle size of 65.36 nm—optimal for nasal mucosa penetration. Studies using Sprague Dawley rats demonstrated the formulation’s superior efficacy compared to traditional delivery routes, with maximum brain concentration reached at 1.5 h post-administration and significantly higher brain uptake compared to oral and IV administration. The nanoemulsion’s effectiveness was attributed to its enhanced drug solubility, protection against biological degradation, and improved nasal mucosa penetration due to its lipophilic nature. In vitro studies using neuroblastoma cells confirmed the formulation’s safety profile, while release studies demonstrated sustained drug delivery [116].

### 6.7. Inorganic NPs

#### 6.7.1. Mimosine Functionalized Gold Nanoparticles

Gold nanoparticles (AuNPs) represent another method for delivering Alzheimer’s therapeutics. These nanoparticles are particularly promising due to their excellent biocompatibility, optical properties, ease of fabrication, and ability to be surface-functionalized with therapeutic compounds [139]. When functionalized with mimosine, a plant-based amino acid with anti-inflammatory and anti-oxidative properties [146], these Au NPs (Mimo-AuNPs) demonstrate significant therapeutic potential. In vitro studies confirm that Mimo-AuNPs efficiently traverse the blood–brain barrier and inhibit both spontaneous and seed-induced aggregation of wild-type and familial mutant human Aβ peptides, as validated by Thioflavin-T (ThT) fluorescence assays. The mechanism involves specific interactions with the hydrophobic domain of Aβ peptides, stabilizing their monomeric conformation and preventing pathological β-sheet formation. Furthermore, it was shown that Mimo-AuNPs can trigger the disassembly of existing Aβ fibrils and confer neuroprotection to mouse cortical neurons against Aβ-mediated toxicity through attenuation of tau protein phosphorylation and the production of harmful oxygen radicals [147]. Studies conducted by Anand et al. showed that Mimo-AuNPs demonstrate a favorable therapeutic window with effective β-amyloid aggregation inhibition starting at 30 μM (50% inhibition) and reaching >90% inhibition at 300 μM, while remaining non-toxic to neurons up to 200 μM, indicating a good safety profile with effective concentrations well within the non-toxic range [139].

#### 6.7.2. Quantum Dots—Caffeic Acid-Coupled Carbon Quantum Dots

In a 2024 study, Hu et al. synthesized a novel biomimetic nanocapsule (CDs-CA-MGs) comprising caffeic acid-coupled carbon quantum dots encapsulated in microglial membranes [140]. Carbon quantum dots are spherical nanostructures that exhibit favorable physicochemical properties including aqueous solubility, surface functionalization capability, and high biocompatibility [148]. The researchers conjugated these quantum dots with caffeic acid (CA), a natural polyphenol with demonstrated anti-inflammatory and antioxidant properties, via boronic acid ester bonds. CA has demonstrated therapeutic efficacy in various neurological disorders, including AD [149]. The microglial membrane encapsulation conferred homologous targeting capabilities, enabling efficient delivery to neuroinflammatory sites following IN administration, thus bypassing the BBB. In vivo studies utilizing 5xFAD transgenic mice demonstrated that CDs-CA-MGs administration significantly attenuated neuroinflammation, reduced neuronal apoptosis, and alleviated cognitive deficits. Mechanistically, the therapeutic effects were mediated through downregulation of pro-inflammatory cytokines (IL-1β, IL-6, TNF-α) and modulation of inflammation-associated signaling pathways, including JAK-STAT and Toll-like receptor cascades [140]. The studies showed that CDs-CA-MGs demonstrate a favorable therapeutic window with the effective anti-inflammatory concentrations at 60 μg/mL being well below cytotoxic levels (>150 μg/mL), providing approximately a 2.5-fold safety margin, and remaining safe up to 1400 μg/mL in hemolysis testing, indicating a wide therapeutic index suitable for safe clinical application [140].

## 7. NPs as Diagnostic Agents: Magnetic Nanoparticles for MRI-Guided Diagnosis in Alzheimer’s Disease

NPs can serve a dual role in Alzheimer’s disease management, functioning not only as therapeutic agents but also as essential components in diagnostic imaging protocols, particularly through their application as MRI contrast agents for early detection of AD pathology. Iron oxide nanoparticles (IONPs) represent a significant advancement over conventional gadolinium-based MRI contrast agents, demonstrating enhanced biocompatibility and reduced toxicity profiles while providing superior targeting specificity for neurological imaging. These magnetic nanoparticles can be functionalized with specific targeting molecules, such as anti-Aβ antibodies, to enable visualization of amyloid structures and tau protein deposits within brain tissue. The superparamagnetic properties of IONPs provide excellent contrast enhancement in MRI studies, while their ability to cross the blood–brain barrier through surface modifications enables effective brain targeting for longitudinal monitoring of disease progression. Furthermore, IONPs conjugated with therapeutic molecules can serve dual functions as both diagnostic tracers and drug delivery systems, representing a theranostic approach that combines imaging capabilities with therapeutic intervention in a single platform. This integration of diagnostic and therapeutic functions makes magnetic nanoparticles particularly valuable tools for neurodegenerative disorders, facilitating early pathology identification, real-time disease progression assessment, and targeted therapeutic delivery through MRI-guided interventional approaches [150,151] [Table 2].

## 8. Conclusions

Nanotechnology presents promising potential use in the treatment of AD. Considering their structure, NPs can be classified into organic (e.g., liposomes, micelles, polymeric), inorganic (e.g., pure metals, metal oxides), or hybrid types. Depending on the type of nanoparticle, its properties vary. It is also possible to modify some of their characteristics to increase their effectiveness. Furthermore, specialized NPs can be designed to be biocompatible with natural systems and build drug delivery systems targeting specific locations.

One of the pieces of evidence of the effectiveness of using NPs is the study of monoclonal antibody IgG4.1 conjugated to iron oxide nanoparticles monodisperse γ-Fe_2_O_3_ nanoparticles as potential treatment for CAA. It proves the ability of the IgG4.1-nanoparticles to target CVA deposits in the brain arterioles. Thus, it can be concluded that targeted NPs make it possible to diagnose and treat cerebral amyloid angiopathy.

Other interesting possibilities for the use of nanotechnology, but many still require research; these include bosentan NPs and NPs combined with angiotensin-converting enzyme inhibitors or angiotensin-receptor blockers in the treatment of cerebral blood flow in AD.

Due to the amazing properties of NPs, such as the ability to surround, transport, protect, and increase the bioavailability of therapeutic agents, it is possible to create nanotechnology-based drug delivery systems through the BBB targeting neuronal delivery, reducing neurodegeneration.

In addition, it is very important to emphasize the anti-inflammatory and neuroprotective effects of nanoparticles. Our review discusses the effects of liposomes, micelles, solid lipid nanoparticles, polymeric nanoparticles, dendrimers, nanoemulsions, and inorganic nanoparticles. For example, the use of metformin encapsulated in phosphatidylserine-based liposomes shows a reduction in neuroinflammation and neuronal necrosis and enhanced neurogenesis. Another example of the unusual action of nanoparticles are ROS-responsive micelles loaded with curcumin, the use of which is directed to the early AD phase even before irreversible damage to neurons. The studies discussed confirm the advantages of using nanoparticles in therapy due to their effectiveness in targeting the causative factors in the pathophysiology of Alzheimer’s disease. Still, further research is required.

## Figures and Tables

**Table 1:** The Summary of the description and characteristics of the systems mentioned in the work and their applications.

NP System	General Characteristic	Possibilities of Appliance
Liposomes	Amphiphilic, spherical, Polar lipids, dispersed in an aqueous solution, Built from phospholipid bilayer membranes, Hydrophobic tails with hydrophilic heads create two different environments, Easy to obtain, A high degree of customization. [24,25,27,28]	Delivery of acetylcholinesterase inhibitors (Galanthamine hydrobromide, Rivastigmine) with enhanced pharmacokinetics and BBB penetration [107,108]. Metformin delivery for reducing neuroinflammation and improving cognitive parameters [109]. Osthole delivery for protection against Aβ oligomer-induced toxicity [110]. ApoE2 gene delivery for enhanced amyloid-beta clearance [111,112]. Pantothenate delivery for inhibiting PKM2 nuclear translocation in microglial cells [113,114].
Micelles	Spherical, amphiphilic, Monolayered structure, The shell is hydrophilic, and the core is hydrophobic, Easily absorbed by cells. [29,31,32]	Targeted delivery of resveratrol to neuronal mitochondria for improving mitochondrial function and reducing amyloid deposition [115,116]. ROS-responsive delivery of curcumin for targeting early-stage AD pathology and microglial abnormality [117,118,119]. Lactoferrin-conjugated delivery of linoleic acid for preventing Aβ peptide oligomerization and tau phosphorylation [120,121,122].
Solid Lipid NPs	Spherical systems estimated up to 1000 nm, Solid lipid matrix with suspended molecules, especially drugs, The layer of surfactant is necessary to stabilize the structure in an aqueous solution, Perfectly compatible with hydrophobic drugs. [33,36]	Delivery of acetylcholinesterase inhibitors (GH, Rivastigmine) with improved bioavailability [120,123]. Erythropoietin delivery for maintaining neuronal survival and reducing oxidative stress [123,124]. Delivery of Lepidium sativum seed bioactive components for neuroprotection against oxidative stress [125].
.Polymeric NPs	Different shapes (e.g., polymeric vessels, nanosphere, dendrimer, polymeric micelles, and polymeric hydrogel), The possibility of being covered with dedicated coats, allowing for targeted transport, High drug-loading capacity, From the building blocks of PNPs, PLGA is one of the most commonly used. [37,38]	PLGA-PEG nanoparticles for curcumin delivery with enhanced brain bioavailability [126]. Redox nanoparticles for ROS scavenging and reducing oxidative damage [127,128]. Epigallocatechin-3-gallate (EGCG) and ascorbic acid co-delivery for enhanced neuroprotection [129,130,131]. Chitosan-based delivery of luteolin for enhanced brain targeting [132,133,134].
Dendrimers	Symmetrical, An atom in the center with the branches spread around, Create a specialized, well-defined spherical 3D structure, High compatibility with the biological system (used as analogs). [40,41]	Lactoferrin-conjugated PAMAM dendrimers for memantine delivery with improved brain bioavailability [135,136]. Targeted delivery of nSMase2 inhibitors for reducing pathological tau spread [137].
Nanoemulsions	Metastable dispersion of nanoscale droplets of one fluid within another fluid, Three main types of emulsions: oil-in-water, water-in-oil, and bicontinuous NEs, Characteristic blue-shining appearance, Prevention of creaming, which means efficient long-term stability, The need for high energy input. [42,43]	Enhanced intranasal delivery of donepezil (acetylcholinesterase inhibitor) with improved brain penetration and reduced gastrointestinal side effects [116,138].
Inorganic NPs (e.g., gold, quantum dots)	Great alternative to organic ones, Rar more flexible and versatile, AuNP acts with a high level of biocompatibility, it is easy to synthesize or bioconjugate, and it indicates great physical properties such as thermostability, QDs have fluorescent and unique optical properties, Significant toxic properties. [47,48]	Mimosine-functionalized gold NPs for inhibiting Aβ aggregation and providing neuroprotection. Therapeutic Window: Effective range: Starting at 30 μM (50% inhibition) up to 300 μM (>90% inhibition). Non-toxic dose: Up to at least 200 μM in neuronal cultures [139]. Caffeic acid-coupled carbon quantum dots for targeting neuroinflammation and reducing neuronal apoptosis. Therapeutic Window: Cell Viability (CCK-8 assay) Safe range: 0–150 µg/mL Hemolysis Testing: Safe up to: 1400 µg/mL. Effective Treatment Concentrations: In Vitro Studies, effective concentration: 60 μg/mL CDs-CA-MGs. In Vivo Studies, effective dose: 20 mg/kg CA equivalent [140].

**Table 2:** Comparative analysis of iron oxide nanoparticles (IONPs) versus conventional gadolinium-based contrast agents for Alzheimer’s disease MRI diagnosis based on [150,151].

Parameter	Conventional Gadolinium-Based Agents	IONPs
BBB Penetration	Limited	Enhanced
Targeting	Non-Selective	Selective AD Biomarker Targeting
Safety Profile	Toxic risks	Superior Safety Profile
Functionality	Diagnostic Only	Diagnostic and therapeutic (drug delivery)
